# A Dual-Sensor-Based Screening System for In Vitro Selection of TDP1 Inhibitors

**DOI:** 10.3390/s21144832

**Published:** 2021-07-15

**Authors:** Ann-Katrine Jakobsen, Josephine Geertsen Keller, María Gonzalez, Endika Martin-Encinas, Francisco Palacios, Concepcion Alonso, Birgitta Ruth Knudsen, Magnus Stougaard

**Affiliations:** 1 Department of Pathology, Aarhus University Hospital, N 8200 Aarhus, Denmark; akj@clin.au.dk; 2 Department of Clinical Medicine, Aarhus University, N 8200 Aarhus, Denmark; jgk@clin.au.dk; 3 Department of Molecular Biology and Genetics, Aarhus University, C 8000 Aarhus, Denmark; brk@mb.au.dk; 4 Department of Organic Chemistry I, Faculty of Pharmacy and Lascaray Research Center, University of Basque Country (UPV/EHU), 01006 Vitoria-Gasteiz, Spain; mariagonzalez@ajlsa.com (M.G.); endika@ipna.csic.es (E.M.-E.); francisco.palacios@ehu.eus (F.P.); concepcion.alonso@ehu.eus (C.A.)

**Keywords:** tyrosyl-DNA phosphodiesterase 1, topoisomerase 1, DNA sensor, inhibitor, cancer, drug screening, biosensor

## Abstract

DNA sensors can be used as robust tools for high-throughput drug screening of small molecules with the potential to inhibit specific enzymes. As enzymes work in complex biological pathways, it is important to screen for both desired and undesired inhibitory effects. We here report a screening system utilizing specific sensors for tyrosyl-DNA phosphodiesterase 1 (TDP1) and topoisomerase 1 (TOP1) activity to screen in vitro for drugs inhibiting TDP1 without affecting TOP1. As the main function of TDP1 is repair of TOP1 cleavage-induced DNA damage, inhibition of TOP1 cleavage could thus reduce the biological effect of the TDP1 drugs. We identified three new drug candidates of the 1,5-naphthyridine and 1,2,3,4-tetrahydroquinolinylphosphine sulfide families. All three TDP1 inhibitors had no effect on TOP1 activity and acted synergistically with the TOP1 poison SN-38 to increase the amount of TOP1 cleavage-induced DNA damage. Further, they promoted cell death even with low dose SN-38, thereby establishing two new classes of TDP1 inhibitors with clinical potential. Thus, we here report a dual-sensor screening approach for in vitro selection of TDP1 drugs and three new TDP1 drug candidates that act synergistically with TOP1 poisons.

## 1. Introduction

Modern small-molecule-based anticancer treatments, especially within precision medicine, are based on targeting specific cellular mechanisms or enzymatic reactions driving the cancer. With the increased knowledge of the biological mechanisms driving different cancer types, specific anticancer targets are identified, and with the large amount of already synthesized small molecular compounds, the modern approach often is to perform a wide screen of small molecule panels for inhibitory effect of the relevant targets. DNA sensors capable of determining the effect of small molecular compounds in screening-based setups are therefore becoming increasingly important for discovery, characterization, and validation of new anticancer drugs [1,2,3].

The DNA-interacting enzyme tyrosyl-DNA phosphodiesterase 1 (TDP1) has, by many, been considered a promising target in anticancer treatment [4,5,6,7,8]. Currently, there is no clinically approved inhibitors on the market; however, several inhibitors have been published [9,10,11,12,13,14,15,16,17,18,19,20]. TDP1 is involved in many different DNA repair processes due to its ability to remove 3′-adducts by catalyzing the hydrolysis of 3′-phosphodiester bonds [21,22,23]. Examples of 3′-adducts repaired by TDP1 are 3′-phosphotyrosine ends, 3′-phosphoglycolate ends, and 3′-deoxyribose phosphate ends. Additionally, several studies have indicated that TDP1 is involved in resolving double-stranded breaks by participation in the non-homologous end joining (NHEJ) repair process [24,25,26]. The relevance for TDP1 targeting in anticancer treatment is supported by different studies finding that TDP1 activity is upregulated from normal to tumor tissue in samples from non-small-cell lung cancer patients [6,27] and that loss of TDP1 sensitizes cancer cells to different anticancer drugs [28,29]. Thus, it is highly relevant to search for TDP1 inhibitors for use in anticancer treatment. However, when searching for TDP1 inhibitors, it is crucial to consider the interference of the inhibitors with the enzyme topoisomerase 1 (TOP1), due to the biological interplay of the two enzymes.

A main function of TDP1 seems to be repair of TOP1 cleavage-induced DNA damage where TOP1 is covalently trapped on the 3′-end of the DNA [7]. Trapping of TOP1 can either occur naturally as a consequence of endogenous DNA damages such as mismatches and abasic sites [30], or it can be induced by using anticancer drugs targeting TOP1 [31]. Both cases result in the generation of TOP1 cleavage complexes (TOP1cc) that, if left unrepaired, are converted into cytotoxic double-stranded DNA breaks upon collision with DNA replication forks [32]. Consequently, anticancer drugs targeting TOP1 are highly toxic for fast dividing cells, such as cancer cells, and are clinically used in treatment of, e.g., colon cancer, ovarian cancer, and small cell lung cancer [33,34,35], where lethal TOP1cc is introduced by analogues of the small molecule, camptothecin (CPT) [36,37,38]. As the anti-cancer effect of CPT and its analogous are strictly dependent on the generation of TOP1cc, any drug preventing TOP1 from cleaving DNA will counteract its cytotoxic effect. Thus, for identification of new TDP1 inhibitors to be used in synergy with TOP1 inhibitors, the effect of the TDP1 inhibitor on the catalytic activity of TOP1 has to be ascertained. Furthermore, many of the published small molecule inhibitors of TDP1 are either dual inhibitors of TDP1 and TOP1 or their effect on TOP1 activity has not been tested [39,40,41,42,43,44,45,46]. The application potentials for dual TDP1 and TOP1 inhibitors may, however, be limited by the high toxicity of targeting TOP1 exemplified by CPT drugs causing multiple side effects [47,48]. Co-treatment with individual TDP1 and TOP1 inhibitors will allow for the lowest possible dosage of each drug, thereby reducing the risk and severity of side effects. Additionally, the development of specific TDP1 inhibitors broadens the field of application due to the various repair capabilities of TDP1 mentioned above.

Although various sensors have been published for measurement of TOP1 [49,50] and TDP1 [51,52,53,54], most in vitro TOP1 and TDP1 drug-screening approaches still use gel-based assays for measuring TDP1 and TOP1 activity [55,56,57]. Although the usability of gel-based assays is well documented, they are time-consuming and not well suited for high-throughput screening. As an alternative to gel-based assay, we have previously developed specific and reliable sensor systems for the measurement of both TDP1 [51] and TOP1 [49] activity. 

Thus, we here report, for the first time, a dual-sensor-based in vitro screening setup for the identification of TDP1 inhibitors that is capable of taking into account the biological interplay between TDP1 and TOP1. We demonstrate that the sensor setup can identify TDP1 inhibitors in vitro that do not affect TOP1 activity, and we confirm that the identified inhibitors act synergistically with TOP1 poisons in vivo even at low doses. Using the sensor-based in vitro screening setup presented in this study, we have identified three new TDP1 inhibitors, NAF-15, PSTHQ-2, and PSTHQ-13, with promising clinical perspectives. 

## 2. Materials and Methods

### 2.1. The Molecular Compounds

**Figure A1:**
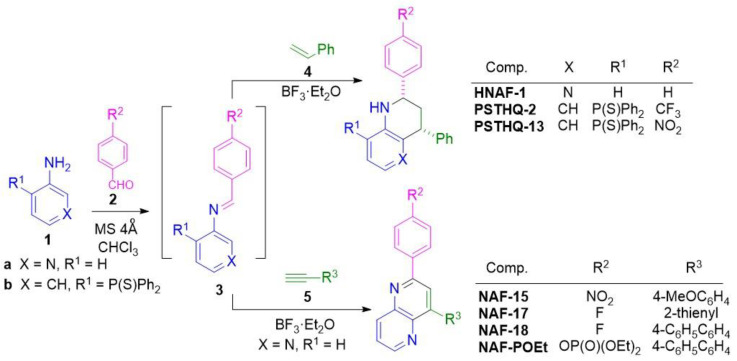
Povarov reaction for the syntheses of HNAF-1, PSTHQ-2, PSTHQ-13, NAF-15, NAF-17, NAF-18, and NAF-POEt.

The synthesis of six of the seven molecular compounds was published previously: HNAF-1 [58], PSTHQ-2 [59], PSTHQ-13 [60], NAF-15 [61], NAF-17 [62], and NAF-18 [62]. General experimental information is described in Tejeria et al. [60], and the synthesis of NAF-POEt is described in Appendix A and Figure A1.

### 2.2. TDP1 In Vitro Activity Assay

In vitro testing of the small molecules’ capability of TDP1 inhibition was carried out by using a fluorescent TDP1 DNA sensor (5′-ATTO488-AAA GCA GGC TTC AAC GCA ACT GTG AAG ATC GCT TGG GTG CGT TGA AGC CTG CTT T-BHQ1-3′, DNA technology) described in P. Jensen et al. [51] and A.-K. Jakobsen et al. [63]. Then, 20 ng of purified human TDP1 (purified as described in Jensen et al. [51]) was incubated with the potential TDP1 inhibitors in a final concentration of 80 µM for each drug or DMSO equivalent to the DMSO content of the potential TDP1 inhibitors (1.6%). After incubation, a final concentration of 0.5 µM TDP1 DNA sensor was added on ice in a buffer containing 20 mM Tris–HCl, pH 8, 100 mM KCl, 10 mM DTT, 10 mM EDTA, and 0.05% Triton X-100. A final volume of 25 µL reaction was transferred to a black 384-well plate, and fluorescence was measured every 30 s for 1 h at 494 nm excitation and 518 nm emission, in a FlexStation^®^ 3 Multi-Mode Microplate Reader, at 37 °C. The initial linear slope was used as a relative measure of TDP1 activity. All measurements were performed in triplicate.

To test if the different groups were statistically significantly different from each other, the means of the two groups were compared by using a paired *t*-test.

### 2.3. IC50

IC50 measurements for the TDP1 inhibitors, NAF-15, PSTHQ-2, and PSTHQ-13, were performed by using the “2.2. TDP1 in vitro activity assay” described above. TDP1 activity was measured after treatment with the following concentrations of TDP1 inhibitor: 0.049, 0.10, 0.20, 0.39, 0.78, 1.56, 3.125, 6.25, 12.5, 25, 50, 100, and 200 µM. The final volume of DMSO was 2% for all TDP1 inhibitors. The “no drug control” contained 2% DMSO. All measurements were performed in triplicate.

The inhibitory effect of the compounds was evaluated by comparing the TDP1 activity at the 13 different concentrations of drug to the no-drug control. The data were analyzed with GraphPad Prism version 8.4.1, using non-linear curve fitting.

### 2.4. TOP1 In Vitro Activity Assay-Rolling-Circle Enhanced Enzyme Activity Detection (REEAD)

SuperEpoxy 2 slides were from Arrayit Coroporation, and vectashield was from Vector Laboratories. Phi29 DNA polymerase was from Thermo-scientific. The following oligonucleotides were used for the experiment: TOP1 substrate (5′-AGA AAA ATT TTT AAA AAA ACT GTG AAG ATC GCT TAT TTT TTT AAA AAT AAA TCT AAG TCT TTT AGA TCC CTC AAT GCA CAT GTT TGG CTC CGA TCT AAA AGA CTT AGA-3′), REEAD primer (5′-/5AmMC6/CCA ACC AAC CAA CCA AAT AAG-3′) and a detection probe (5′-6FAM-CCT CAA TGC ACA TGT TTG GCT CC-3′). All oligonucleotides were synthesized by DNA Technology A/S, Aarhus, Denmark.

The REEAD assay was carried out as previously described in Stougaard et al. [49]. Briefly, SuperEpoxy2 slides were coupled with 10 µM REEAD primer specific for the TOP1 substrate in 300 mM Na_3_PO_4_, pH 8.5, and incubated overnight in a humidity chamber with saturated NaCl. The slides were blocked at 50 °C in blocking buffer (50 mM Tris, 50 mM Tris-HCl, and 32 mM ethanolamine, pH 9), followed by wash in wash buffer 1 (4× SSC and 0.1% SDS).

The TOP1 reaction was carried out in TOP1 buffer (10 mM Tris-HCl, pH 7.5, 5 mM CaCl_2_, 5 mM MgCl_2_, 10 mM DTT, and 0.2 µg/µL BSA) supplemented with 0.5 M NaCl and purified TOP1 (purified as described in Lisby et al. [16]). DMSO, 80 µM TDP1 inhibitor (NAF-15, PSTHQ-2, or PSTHQ-13), or CPT was added, and the samples were incubated for 30 min at 37 °C. Reactions were initiated by addition of 1 µM TOP1 substrate and incubated for 10 min at 20 °C. The reactions were stopped with 0.2% weight volume 1% SDS. The generated TOP1 circles were hybridized to the activated Epoxy slides for 1 h at 37 °C.

Rolling-circle amplification was carried out in 1× Phi29 buffer supplemented with 0.2 µg/µL BSA, 1 unit/µL phi29 polymerase, and 1 mM dNTP. The slides were incubated for 1 h at 37 °C and subsequently washed for 1 min at 20 °C in wash buffer 2 (100 mM Tris-HCl, 150 mM NaCl, and 0.3% SDS), followed by 1 min wash in wash buffer 3 (100 mM Tris-HCl, 150 mM NaCl, and 0.05% Tween20). Finally, the slides were dehydrated in 96% in EtOH for 1 min.

The generated rolling-circle products were visualized by hybridization of 200 nM fluorescent detection probe (FAM labeled) to the rolling-circle products in hybridization buffer (20% formamide, 2× SSC, and 5% glycerol) for 30 min at 37 °C. The slides were washed in wash buffer 2 and 3, followed by dehydration in EtOH. The slides were mounted with Vectashield without DAPI and then visualized by using 60× objective in a fluorescent microscope (Olympus IX73). Signals detected in an average of 12 microscopic images were counted in ImageJ, and all data were plotted as mean with standard deviation. All measurements were performed in triplicate.

### 2.5. Cell Culture

HeLa cells were cultured in Dulbecco’s Modified Eagle’s Medium supplemented with 10% heat-inactivated fetal bovine serum (FBS) and 1% Penicillin–Streptomycin. All reagents were from Gibco, ThermoFisher (Waltham, MA, USA).

### 2.6. Cell Survival after Co-Treatment of TDP1 Drugs and SN-38

To test for survival of HeLa cells after treatment with TDP1 inhibitors and/or SN-38, HeLa cells were seeded in a quantity of 5000 cells/well in 100 µL culture media in a 96-well culture plate. The following day, the cells were treated with DMSO alone (0.5%), TDP1 inhibitor alone, SN-38 alone, or a combination of TDP1 inhibitor and SN-38. The final concentration of SN-38 was 10 nM, and the final concentration of TDP1 inhibitor was 17.5 µM for NAF-15 and 35 µM for PSTHQ-2 and PSTHQ-13. After 72 h of drug treatment, cell survival was measured by using PrestoBlue Cell Viability Reagent (ThermoFisher). Each well was treated with 10 µL PrestoBlue and incubated at 37 °C and 5% CO_2_ for 30 min, before fluorescence was measured at 560 nm excitation and 590 nm emission in a FlexStation^®^ 3 Multi-Mode Microplate Reader. The mean of fluorescence from 12 wells without cells was subtracted from the results in order to correct for background. Twelve different wells were treated with the same drug conditions.

To test if the different groups were statistically significantly different from each other, the means of the two groups were compared by using a paired *t*-test.

### 2.7. Cell Survival with Titration of Potential TDP1 Inhibitor

The same procedure as for “2.6. Cell survival after co-treatment of TDP1 drugs and SN-38” was used to test different concentrations of TDP1 inhibitors in combination with 10 nM SN-38. For NAF-15, the concentrations 0.05, 0.1, 1, 5, 10, and 25 µM were tested, and an NAF-15 control (25 µM) without SN-38 was included. For PSTHQ-2 and PSTHQ-13, the concentrations 0.1, 1, 5, 10, 25, and 50 µM were tested, and a PSTHQ-2 or PSTHQ-13 control (50 µM) without SN-38 was included. One SN-38 control with 10 nM SN-38 was also included. The final volume of DMSO in the DMSO controls and drug samples was 0.5%.

To test if the different groups were statistically significantly different from each other, the means of the two groups were compared by using a paired *t*-test.

## 3. Results

### 3.1. Structure of Potential TDP1 Inhibitors

**Figure 1:**
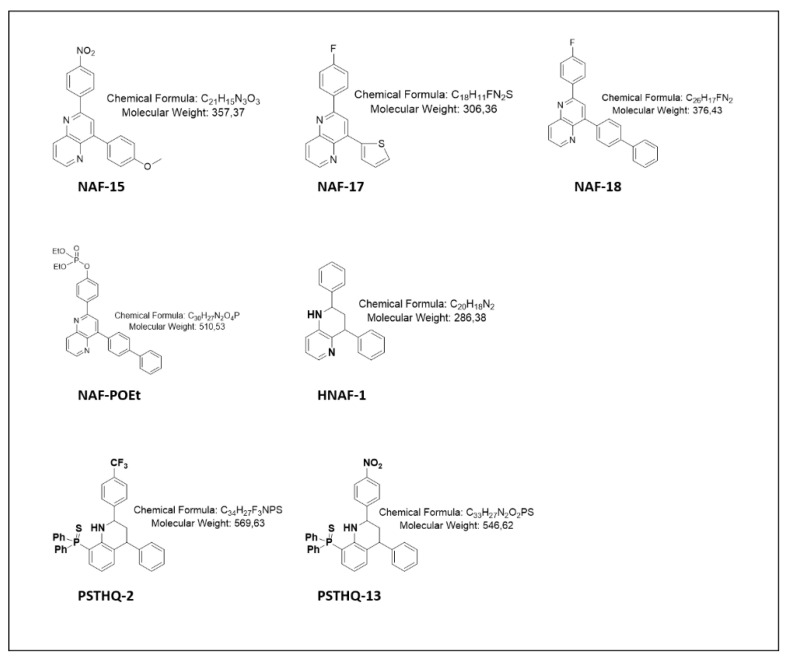
Molecular structure, chemical formula and molecular weight for the seven small compounds designed to inhibit the enzymatic activity of TDP1.

We have, in an unpublished study, screened 40 small molecular compounds for inhibitory effect on TDP1 activity to select the best compounds for this study. The 40 compounds were previously developed and investigated for TOP1 inhibitory effect or antileishmanial activity. Based on the structural comparison of the most potent TDP1 inhibitors among the 40 compounds, seven small molecular compounds were selected from a library of compounds developed at Department of Organic Chemistry I, University of Basque Country (UPV/EHU), and resynthesized for investigation in this study. The molecular structures, chemical formula, and molecular weight of the seven compounds are presented in Figure 1.

### 3.2. In Vitro Inhibition of TDP1 and TOP1 Enzyme Activity with NAF-15, PSTHQ-2, and PSTHQ-13

**Figure 2:**
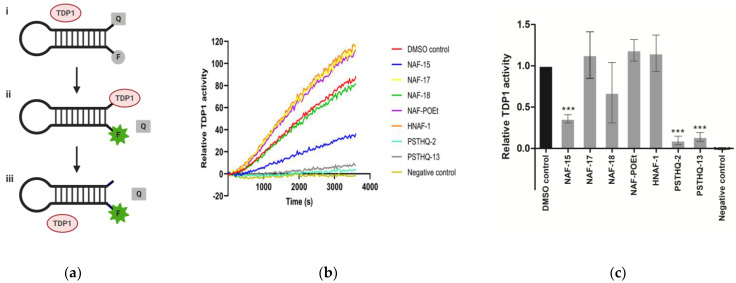
TDP1 activity after treatment with molecular compounds. (**a**) Illustration of the TDP1 activity assay using a hairpin DNA substrate with a quencher (Q: BHQ-1) in the 3′-end and a fluorophore (F: ATTO488) in the 5′-end (i). TDP1 removes the quencher (ii) followed by release of the TDP1 protein, now capable of participating in a new cycle (iii). Removal of the quencher allows for light development of the fluorophore. Fluorescence development is measured over time. (**b**) Example of a curve representing measurement of fluorescence development over time. The slope of the initial linear phase is a representation of the TDP1 activity assay visualized as a columns chart in c. (**c**) TDP1 activity for purified TDP1 enzyme after treatment with potential TDP1 inhibitors. All potential TDP1 inhibitors had a final concentration of 80 µM. DMSO control contained 1.6% DMSO, which is the same DMSO amount as in the samples with potential TDP1 inhibitors. All data were normalized to DMSO control. *** Represents a *p*-value < 0.001 compared to the DMSO control.

In order to investigate the inhibitory effect of the compounds NAF-15, NAF-17, NAF-18, NAF-POEt, HNAF-1, PSTHQ-2, and PSTHQ-13 (Figure 1) on TDP1 enzymatic activity, we used a DNA sensor previously developed by our group and designed to measure the ability of TDP1 to remove 3′-adducts [51]. The DNA sensor is composed of a DNA oligonucleotide that fold into a hairpin structure having a fluorophore at the 5′-end and quencher at the 3′-end (Figure 2a). TDP1 mediated hydrolysis removes the quencher, thereby enabling light emission from the fluorophore by which TDP1 activity can be measured as fluorescent development over time. In the experimental setup, we measured the activity of 20 ng of purified TDP1 in terms of fluorescent emission generated in the absence or presence of 80 µM of each of the molecular compounds, as shown in Figure 2b. The relative TDP1 activity after treatment with the small molecular compounds was calculated as the slope of the initial linear phase of florescence development and presented in Figure 2c. As seen from Figure 2c, addition of NAF-15, PSTHQ-2, and PSTHQ-13 resulted in a statistically significant inhibition of TDP1 activity when comparing to the DMSO control. DMSO was used as a control since all compounds were dissolved in DMSO. NAF-15, PSTHQ-2, and PSTHQ-13 reduced the TDP1 activity with 64.1%, 90.4%, and 86.1%, respectively. The IC50 value for TDP1 inhibition was measured for all three compounds by incubating the TDP1 sensor with purified TDP1 and 13 different concentrations of compounds or with DMSO. Based on the results from these experiments, the IC50 values were calculated to be 37.8 µM for NAF-15, 4.28 µM for PSTHQ-2, and 13.1 µM for PSTHQ-13 (Table 1).

The four compounds, NAF-17, NAF-18, NAF-POEt, and HNAF, did not produce a significant decrease in TDP1 activity and were not included in any further experiments.

**Figure 3:**
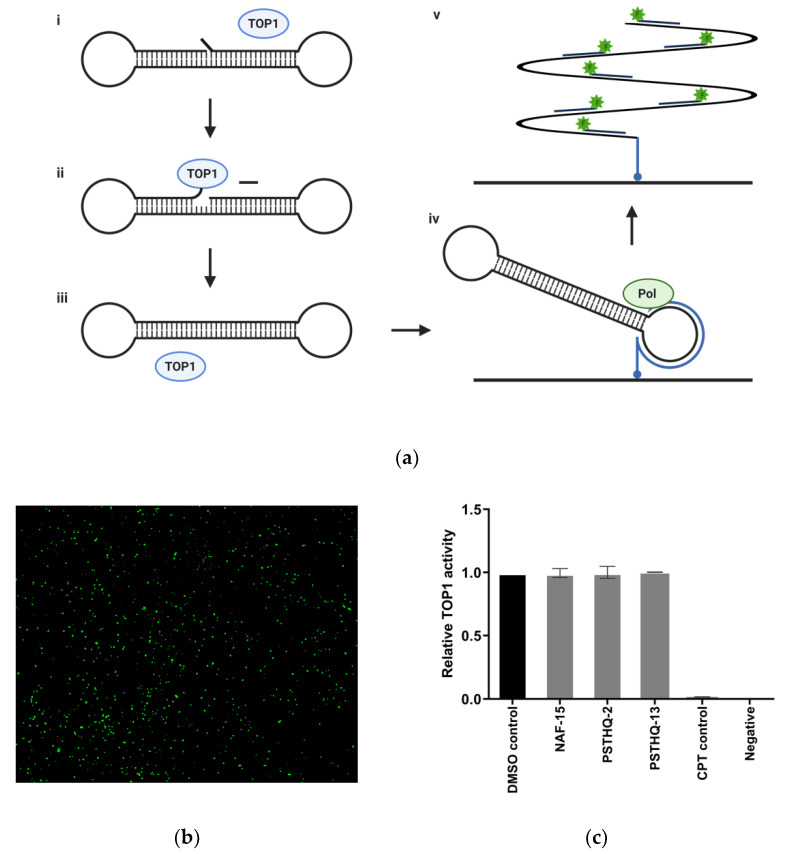
TOP1 activity after treatment with molecular compounds. (**a**) Illustration of the TOP1 activity assay named Rolling-circle Enhanced Enzyme Activity Detection (REEAD). The TOP1 sensor is a dumbbell DNA substrate with an AGA 5′overhang (i). TOP1 cleaves the AGA overhang (ii) and ligates the remaining nick, thereby releasing the TOP1 protein capable of participating in a new cycle and leaving a closed dumbbell substrate (iii). The primer for rolling circle amplification (RCA) is coupled to a microscopic slide, and the dumbbell substrate is hybridized to the RCA primer. Phi29 polymerase (Pol) initiates RCA from the RCA-primer, using the closed dumbbell substrate as template (iv). Fluorophore (F) coupled oligoes complementary to the RCA product hybridize to the synthesized strand, making visualization of the RCA-product possible in a fluorescence microscope (v). (**b**) Representative pictures of circles on a microscopic slide where each green dot represents a signal from one dumbbell substrate and thereby one TOP1 cleavage-ligation reaction. Amount of green signals is a measure of TOP1 activity represented as a column graph in c. (**c**) TOP1 activity after treating purified TOP1 enzyme with the three TDP1 inhibitors or CPT. All samples with TDP1 inhibitors and CPT had a final concentration of 80 µM. DMSO control contained 1.6% DMSO, which is the same DMSO amount as in the samples with potential TDP1 inhibitors and CPT. CPT control is a positive control for TOP1 inhibition. All data were normalized to the DMSO control.

To investigate if the compounds had any effect on TOP1 activity, we tested them by using the REEAD assay illustrated in Figure 3a,b for measuring the TOP1 cleavage-ligation activity, which has previously been described by Stougaard et al. [49,64]. TOP1 activity was measured in the presence of purified TOP1 and NAF-15, PSTHQ-2, PSTHQ-13, CPT, or DMSO. The results are depicted in Figure 3c. As evident from Figure 3c, TOP1 activity was not affected by any of the compounds when comparing to the DMSO control.

### 3.3. Cell Survival in Presence of NAF-15, PSTHQ-2, and PSTHQ-13

**Figure 4:**
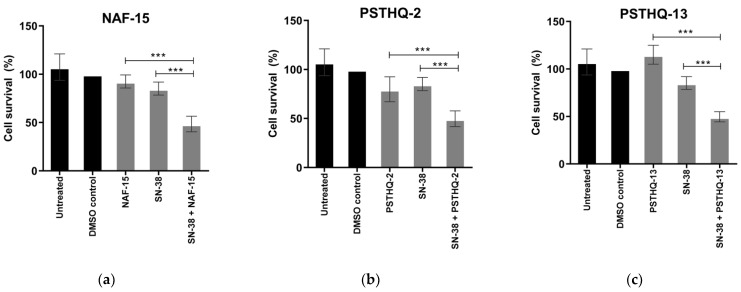
Cell survival after treatment with the TDP1 inhibitors NAF-15 (**a**), PSTHQ-2 (**b**), and PSTHQ-13 (**c**) in combination with the TOP1 poison, SN-38, for 72 h, in HeLa cells. All SN-38 containing samples contained 10 nM SN-38. DMSO control contained 0.5% DMSO, which is the same DMSO amount as in all drug containing samples. The TDP1 inhibitors have a final concentration of (**a**) 17.5 µM NAF-15, (**b**) 35 µM PSTHQ-2, and (**c**) 35 µM PSTHQ-13. Cell survival was measured by using PrestoBlue. All data were normalized to the DMSO control. *** Represents a *p*-value < 0.001.

To examine the cytotoxic effect of the three molecular compounds capable of inhibiting TDP1, HeLa cells were treated with NAF-15, PSTHQ-2, or PSTHQ-13 for 72 h. The final concentration of the molecular compounds was 17.5 µM for NAF-15 and 35 µM for PSTHQ-2 and PSTHQ-13, which resulted in the highest possible concentration of compound without exceeding a final concentration of 0.5% DMSO. Cell survival after 72 h treatment with NAF-15, PSTHQ-2, and PSTHQ-13 is depicted in Figure 4. No significant decrease in cell survival was seen for NAF-15 (Figure 4a), PSTHQ-2 (Figure 4b), or PSTHQ-13 (Figure 4c) when compared to the DMSO control, which is the solvent of the compounds.

To investigate the molecular compounds’ ability to decrease cell survival when combined with a TOP1 poison, HeLa cells were treated with NAF-15, PSTHQ-2, or PSTHQ-13 in combination with a very low dose of SN-38, the active metabolite of the clinically applied CPT analog, Irinotecan. To increase the possibility of detecting a potential synthetic lethality of TDP1 and TOP1 inhibition, a concentration of SN-38 resulting in a very low degree of cytotoxicity in HeLa cells was used for the experiment. Cell survival after 72 h of treatment with NAF-15, PSTHQ-2, or PSTHQ-13 in combination with SN-38 are presented in Figure 4. NAF-15 (Figure 4a), PSTHQ-2 (Figure 4b), and PSTHQ-13 (Figure 4c) all decreased cell survival when co-treated with a low dose of SN-38 when compared to treatment with NAF-15, PSTHQ-2, PSTHQ-13, or SN-38 alone.

**Figure 5:**
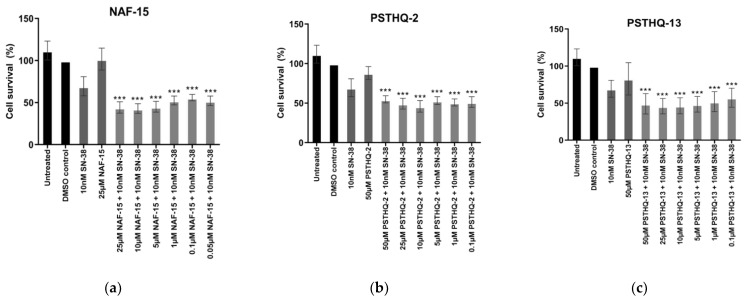
Cell survival after treatment with different concentrations of the TDP1 inhibitors NAF-15 (**a**), PSTHQ-2 (**b**), and PSTHQ-13 (**c**) in combination with the TOP1 poison, SN-38, for 72 h, in HeLa cells. All SN-38 containing samples contain 10 nM SN-38. DMSO control contained 0.5% DMSO, which is the same DMSO amount as in all drug-containing samples. The TDP1 inhibitors had a concentration range of 0.05 to 25 µM for NAF-15 (**a**) and 0.1 to 50 µM for PSTHQ-2 (**b**) and PSTHQ-13 (**c**). Cell survival was measured by using PrestoBlue. All data were normalized to DMSO control. *** Represents a *p*-value < 0.001 compared to TDP1 inhibitor or SN-38 alone.

To test if low doses of NAF-15, PSTHQ-2, and PSTHQ-13 can induce cell death when co-treated with the low dose of SN-38 initially used, HeLa cells where treated with 10 nM SN-38 and from 0.05 to 25 µM NAF-15 or 0.1 to 50 µM PSTHQ-2/PSTHQ-13. Interestingly, all concentrations of NAF-15 (Figure 5a), PSTHQ-2 (Figure 5b), and PSTHQ-13 (Figure 5c) in combination with a low dose of SN-38 reduced cell survival significantly when compared to treatment with NAF-15, PSTHQ-2, PSTHQ-13, or SN-38 alone. No drug concentrations below 0.05 µM for NAF-15 and 0.1 µM for PSTHQ-2 and PSTHQ-13 were tested.

## 4. Discussion

In this study, we reported a dual-sensor-based in vitro screening system for identification and validation of new TDP1 inhibitors that take into account the biological interplay between TOP1 and TDP1. The sensor system is based on two previously published DNA sensors with a fluorescent readout and measures the small molecular compounds’ effect on the activity of TDP1 and TOP1. We demonstrated the use of the in vitro sensor system for screening and validation of new small molecular compounds with the ability to inhibit TDP1 activity without inhibiting TOP1 activity and validated the in vitro results *in vivo*.

It is of great importance to measure the inhibitory effect of potential TDP1 inhibitors on both TDP1 and TOP1 activity to ensure that the compounds inhibit only TDP1 activity and not TOP1 catalytic activity. The most well-documented application for TDP1 inhibitors is in combination with TOP1 targeting anticancer drugs [28,65,66]. In relation to this, it is important to realize that the TOP1 targeting anticancer drugs are TOP1 poisons stabilizing the transient TOP1–DNA intermediate in the TOP1 catalytic cycle [37]. This will eventually result in cytotoxic double-strand breaks in dividing cells, such as cancer cells [32]. If the potential TDP1 inhibitor blocks the catalytic activity of TOP1, TOP1 poisons cannot exert their effect. Thus, the advantage of using the TDP1 inhibitor to enhance the effect of TOP1 poisons will be lost. Therefore, the concept of testing potential TDP1 inhibitors for effect on TOP1 catalytic activity is crucial. Despite this, most published TDP1 inhibitors are not tested for inhibitory effect on TOP1 activity; however, this can easily be accomplished by using a DNA sensor, as presented in Figure 3.

Initially, 40 small molecular compounds from different studies investigating TOP1 inhibitory effect or antileishmanial activity were screened for their TDP1 inhibitory effect, using the TDP1 sensor (data not shown). Based on structural comparisons of the compounds with the strongest TDP1 inhibition, seven small molecular compounds were selected from a large library of compounds to be used for validation of the dual-sensor-based in vitro screening system. The seven small molecular compounds were, in this study, used to validate our new dual-sensor-based in vitro screening setup. Thus, they were tested in vitro for their ability to inhibit the activities of TDP1 and TOP1. Of these, three compounds, NAF-15, PSTHQ-2, and PSTHQ-13, all showed a high degree of TDP1 inhibition (Figure 2). No inhibitions of TOP1 were detected (Figure 3), thereby proving the capability of the in vitro screening system for the identification of molecular compounds capable of inhibiting TDP1 without influencing the TOP1 activity. To address the performance of the three compounds, the IC50 values for TDP1 inhibition were investigated by using the TDP1 sensor and calculated to be 37.8 µM for NAF-15, 4.28 µM for PSTHQ-2, and 13.1 µM for PSTHQ-13 (Table 1). This is within the normal range compared to other TDP1-inhibiting compounds [67].

Development of compounds that specifically inhibit TDP1 without also inhibiting TOP1 will, in anticancer treatment, allow for more diverse treatment than dual inhibitors, since TDP1 is important in other repair processes than the repair of TOP1cc. So far, TDP1 has been found to participate in the repair of a wide range of 3′-lesions, such as 3′-deoxyribose phosphate ends, 3′-phosphoglycolate ends, and 3′-abasic sites [21,22,68]. Furthermore, several studies have indicated that TDP1 is involved in resolving double-stranded breaks by participation in the NHEJ repair process [24,25,26]. Additionally, TDP1 has been suggested as a promising target in the treatment of HPV-induced cancers, where it, along with PARP1, has been shown to be essential for the initial amplification of the high-risk HPV genome [69]. Because of the various repair functions of TDP1—and with the potential of more being found—the enzyme could prove to be an extremely interesting target in repair deficient tumors. If a tumor is repair-deficient in other repair processes than the ones involving TDP1, inhibition of TDP1 might be beneficial to achieve synthetic lethality and thereby increase cytotoxicity of tumor cells. Treating repair-deficient tumors with TDP1 inhibitors will most likely keep the toxicity and side effects of anticancer treatment at a minimum, since the non-tumor cells in the body will not be repair-deficient and presumably more resistant to drug treatment. Furthermore, toxicity and side effects are expected to be low with the TDP1 inhibitors developed in this study, as they have no cytotoxicity by themselves, even at high concentrations (Figure 4 and Figure 5). The concept of synthetic lethality of repair enzymes has already been applied clinically with great success in the anticancer treatment of BRCA-deficient ovarian tumors treated with PARP1 inhibitors [70]. Interestingly, a combination of TDP1 knockdown and PARP1 inhibition has been shown to be cytotoxic in rhabdomyosarcoma cells even without BRCA alterations [8]. As the cytotoxic effects were preferential for rhabdomyosarcoma cells over those of control myoblasts, the authors hypothesized that this effect is due to the rhabdomyosarcoma cells having repair deficiencies that have to be compensated for by PARP and TDP1. This, combined with TDP1s involvement in many different repair mechanisms, suggests that TDP1 drugs may be useful in many other settings than combined TDP1/TOP1 inhibition.

Even though TDP1 drugs have many potentials besides combined TDP1/TOP1 inhibition, it is still of great importance that the TDP1 drugs can be used in combination with TOP1 poisons of the CPT family. These are already used clinically but have many side effects and are usually administered in low doses, in combination with other drugs, to reduce side effects [71]. Thus, we hypothesized that, if the TDP1 drug could act synergistically with TOP1 poisons, it could enable the use of low-dose TOP1 poison. Therefore, the three TDP1-inhibiting molecules tested in this study, NAF-15, PSTHQ-2, and PSTHQ-13, were tested in combination with the TOP1 poison, SN-38. All three compounds decreased cell survival significantly when combined with a low dose of SN-38 (Figure 4). Furthermore, PSTHQ-2 and PSTHQ-13 were found to reduce cell survival significantly, with a concentration down to 100 nM (no lower concentrations were tested) in combination with a low dose of SN-38 (Figure 5). The same results were observed for NAF-15 with a concentration down to 50 nM (no lower concentrations were tested). This indicates that low doses of all three TDP1 inhibitors can kill cancer cells when combined with a low dose of TOP1 poison. This is very promising for their usability in cancer treatment, where low doses of drugs are preferred to keep the side effects and toxicity of chemotherapeutics at a minimum.

Several small molecular compounds with the ability to inhibit TDP1 activity have been published [9,10,11,12,13,14,15,16,17,18,19,20]. However, most compounds have not been tested in vivo and are only tested in vitro for inhibition of TDP1 (not inhibition of TOP1) [10,12,17,20,72,73,74]. When comparing data for other TDP1 inhibitors tested in vivo in cell cultures [18,75,76,77,78,79,80,81,82] to data in this study, it is noticeable that our TDP1 inhibitors have a good effect on reducing cell survival even at low concentrations of both TDP1 inhibitor and TOP1 poison. In conclusion, the TDP1 inhibitors, namely NAF-15, PSTHQ-2, and PSTHQ-13, identified in this study show promising perspectives for further testing of clinical utility.

Thus, we here reported, for the first time, a sensor based in vitro screening setup for identification of TDP1 inhibitors capable of taking into account the biological interplay between TDP1 and TOP1. We demonstrated that the sensor system can identify TDP1 inhibitors in vitro that do not affect TOP1 activity, and we confirmed that the identified inhibitors act synergistically with TOP1 poisons in vivo even at low doses. Using the sensor based in vitro screening setup presented in this study, we identified three new TDP1 inhibitors, namely NAF-15, PSTHQ-2, and PSTHQ-13, with promising clinical perspectives.

## Figures and Tables

**Table 1:** IC50 values for the three compounds with significant inhibitions of TDP1 activity.

Compound Name	IC50
NAF-15	37.8 µM
PSTHQ-2	4.28 µM
PSTHQ-13	13.1 µM

## Data Availability

Not applicable.

## Appendix A

### Appendix A.1. Chemical Synthesis of Heterocyclic Compounds and Experimental Data of NAF-POEt

Chemical synthesis of heterocyclic compounds are described in Tejeria et al. [60].

4-(4-([1,1′-Biphenyl]-4-yl)-1,5-naphthyridin-2-yl)phenyl diethyl phosphate **(NAF-POEt).** To a solution of 3-aminopyridine **1a** (1 mmol, 94.1 mg) in CHCl_3_ (10 mL) diethyl (4-formylphenyl) phosphate **2a [83]** (1 mmol, 258 mg) was added. The mixture was stirred under a nitrogen atmosphere, at room temperature, for 15 h, and then 4-ethynyl-1,1′-biphenyl **5a** (R^3^ = 4-C_6_H_5_-C_6_H_4_, 1 mmol, 178.1 mg) and BF_3_·Et_2_O (2 mmol, 0.246 mL) were added. The mixture was stirred at reflux until TLC and ^1^H NMR spectroscopy indicated the disappearance of aldimine. The reaction mixture was washed with 2M aqueous solution of NaOH (25 mL) and water (25 mL), extracted with dichloromethane (2 × 25 mL), and dried over anhydrous MgSO_4_. Removal of the solvent under vacuum afforded an oil that was purified by silica gel flash column chromatography, using a gradient elution of 10–40% ethyl acetate in hexane, to afford product **NAF-POEt** (306.1 mg, 60%) as a white solid; mp 121 to 122 ºC (ethyl acetate/hexane). ^1^H NMR (400 MHz, CDCl_3_) δ: 8.90 (dd, ^3^*J*_HH_ = 4.0 Hz, ^3^*J*_HH_ = 1.6 Hz, 1 H), 8.40 (dd, ^3^*J*_HH_ = 8.4 Hz, ^3^*J*_HH_ = 1.6 Hz, 1 H), 8.42–6.76 (m, 14 H), 6.74 (d, ^3^*J*_HH_ = 1.6 Hz, 1 H), 4.19–4.13 (m, 4 H), 1.31–1.29 (m, 6H) ppm. ^13^C {^1^H} NMR (100 MHz, CDCl_3_) δ: 16.1 (d, ^2^*J*_CP_ = 7.0 Hz, CH_3_), 64.7 (d, ^2^*J*_CP_ = 6.0 Hz, CH_2_), 118.6–156.7 (CH, C) ppm. ^31^P NMR (120 MHz, CDCl_3_) δ: -6.31 ppm. HRMS (EI): calculated for C_30_H_27_N_2_O_4_P [M]+ 510.5302 found 510.5299.

### Appendix A.2. Overview

The heterocyclic compounds were prepared by a Povarov reaction between corresponding aldimines **3**, prepared in situ by condensation between aromatic amines **1** and aromatic aldehydes **2**, and dienophiles **4** or **5** (Figure A1). For the preparation of the derivatives **HNAF-1** (X = N, R1 = H), **PSTHQ-2** (X = CH, R1 = P(S)Ph_2_), and **PSTHQ-13** (X = CH, R1 = P(S)Ph_2_), to the aldimine **3** solution an equimolecular amount of styrene **4** was added and the mixture was stirred in refluxing chloroform in the presence of the Lewis acid (BF_3_·Et_2_O) affording the corresponding derivatives **HNAF-1 [58]**, **PSTHQ-2 [59]**, and PSTHQ-13 [60] regio- and stereoselectively as previously reported. For preparation of the derivatives **NAF-15 [61]**, **NAF-17 [62]**, **NAF-18 [62]**, and **NAF-POEt**, aldimines **3** were prepared from amine **1a** (X = N, R^1^ = H) and reacted in situ with an equimolecular amount of the corresponding acetylenic derivative **5** in the presence of BF_3_·Et_2_O in chloroform at reflux, and the corresponding heterocyclic derivatives were obtained regioselectively.
